# Role of extracellular calcium and mitochondrial oxygen species in psychosine-induced oligodendrocyte cell death

**DOI:** 10.1038/cddis.2014.483

**Published:** 2014-11-20

**Authors:** V Voccoli, I Tonazzini, G Signore, M Caleo, M Cecchini

**Affiliations:** 1 NEST, Istituto Nanoscienze-CNR and Scuola Normale Superiore, Piazza San Silvestro 12, 56127 Pisa, Italy; 2 Center for Nanotechnology Innovation@NEST, Istituto Italiano di Tecnologia, Pisa, Italy; 3 CNR Neuroscience Institute, via G. Moruzzi 1, 56124 Pisa, Italy

## Abstract

Globoid cell leukodystrophy (GLD) is a metabolic disease caused by mutations in the galactocerebrosidase (GALC) gene. GALC is a lysosomal enzyme whose function is to degrade galacto-lipids, including galactosyl-ceramide and galactosyl-sphingosine (psychosine, PSY). GALC loss of function causes progressive intracellular accumulation of PSY. It is widely held that PSY is the main trigger for the degeneration of myelinating cells and progressive white-matter loss. However, still little is known about the molecular mechanisms by which PSY imparts toxicity. Here, we address the role of calcium dynamics during PSY-induced cell death. Using the human oligodendrocyte cell line MO3.13, we report that cell death by PSY is accompanied by robust cytosolic and mitochondrial calcium (Ca^2+^) elevations, and by mitochondrial reactive oxygen species (ROS) production. Importantly, we demonstrate that the reduction of extracellular calcium content by the chelating agent ethylenediaminetetraacetic acid can decrease intra-mitochondrial ROS production and enhance cell viability. Antioxidant administration also reduces mitochondrial ROS production and cell loss, but this treatment does not synergize with Ca^2+^ chelation. Our results disclose novel intracellular pathways involved in PSY-induced death that may be exploited for therapeutic purposes to delay GLD onset and/or slow down its progression.

Globoid cell leukodystrophy (GLD), also known as Krabbe disease, is a childhood leukodystrophy triggered by mutations in the galactocerebrosidase (GALC) gene; the physio-pathological hallmarks of GLD are progressive demyelination, reactive astrocytosis and microgliosis.^1^ GALC is a lysosomal enzyme essential for the normal catabolism of galacto-lipids, including galactosyl-ceramide and galactosyl-sphingosine (psychosine, PSY). GALC loss of function causes progressive accumulation of PSY, a cytotoxic metabolite that has been assumed as the main cause for GLD pathogenesis.^2^ PSY leads to Schwann cell and oligodendrocyte death, but still little is known about the molecular mechanisms by which PSY imparts toxicity. It has been demonstrated that PSY accumulates in cell membrane raft micro-domains, disrupting their architecture^3^ and inhibiting protein kinase C translocation to the plasma membrane.^4^ Recently, increased raft clustering was also reported in cultured dorsal root ganglion neurons prepared from the GLD murine model (i.e., the Twitcher mouse), and this was associated with the dysregulation of tyrosine kinase receptor A membrane recruitment and ligand-tyrosine kinase receptor A activated endocytosis.^5^ PSY induces p53-mediated apoptotic cell death,^6^ tumor necrosis factor-related apoptosis,^6, 7^ activation of secretory phospholipase A2,^8^ cytochrome C release from mitochondria and apoptosis activation via the caspase-9 pathway.^9^ Moreover, several authors found that peroxisomal *β*-oxidation was significantly inhibited and very long-chain fatty acid levels and reactive oxygen species (ROS) production were increased in PSY-treated cells.^10, 11^

Calcium (Ca^2+^) is an essential ion for cell life, acting as a key second messenger in almost all cellular functions. It is well established that Ca^2+^ is one of the main second messengers involved in apoptotic cell death in neurons and in other cell types; sustained cytosolic Ca^2+^ increase can activate apoptosis.^12^ This can originate from extracellular influx or by release from intracellular stores like the endoplasmic reticulum.^13^ Importantly, mitochondria are also involved in Ca^2+^ homeostasis.^14^ Mitochondrial Ca^2+^ in basal conditions is maintained at low concentrations, but mitochondria are organelles that can take up high Ca^2+^ concentrations; indeed, different stimuli, such as nutrients, hormones or neurotransmitters that increase the cytoplasmic Ca^2+^ content also induce intra-mitochondrial Ca^2+^ increase.^15^ If this increase is relevant, ROS production increases and this is associated with mitochondrial membrane destruction, release of cytochrome C and apoptosis induction.^16^ During this process, pro-apoptotic Bcl2 family of proteins plays a crucial role by regulating the intracellular/mitochondrial Ca^2+^ content, and by inducing mitochondrial permeabilization, the essential step for cytochrome C release and caspase activation.^12, 17^

It has been reported that some sphingolipid metabolites, such as ceramides and sphingosine, can play a crucial role in many steps of apoptosis induction as regulators of some Bcl2 family proteins, by increasing intracellular Ca^2+^ levels and inducing mitochondrial stress.^18^ However, these mechanisms have never been explored during PSY-induced cell death.

In this article, we report on the role of intracellular Ca^2+^ dynamics during PSY-induced cell death *in vitro.* Using the human oligodendrocyte cell line MO3.13 and fluorescent probes, we measured Ca^2+^ variations in cytoplasm and mitochondria upon PSY administration until cell death. Moreover, we studied oxidative stress production in mitochondria by flow cytometry and time-lapse confocal fluorescence microscopy. Finally, in order to rescue cell viability in presence of PSY, we investigated the use of Ca^2+^ chelation in the extracellular medium, and its possible synergic effect with antioxidant treatment.

## Results

### PSY induces apoptotic and necrotic cell death

**Figure 1:**
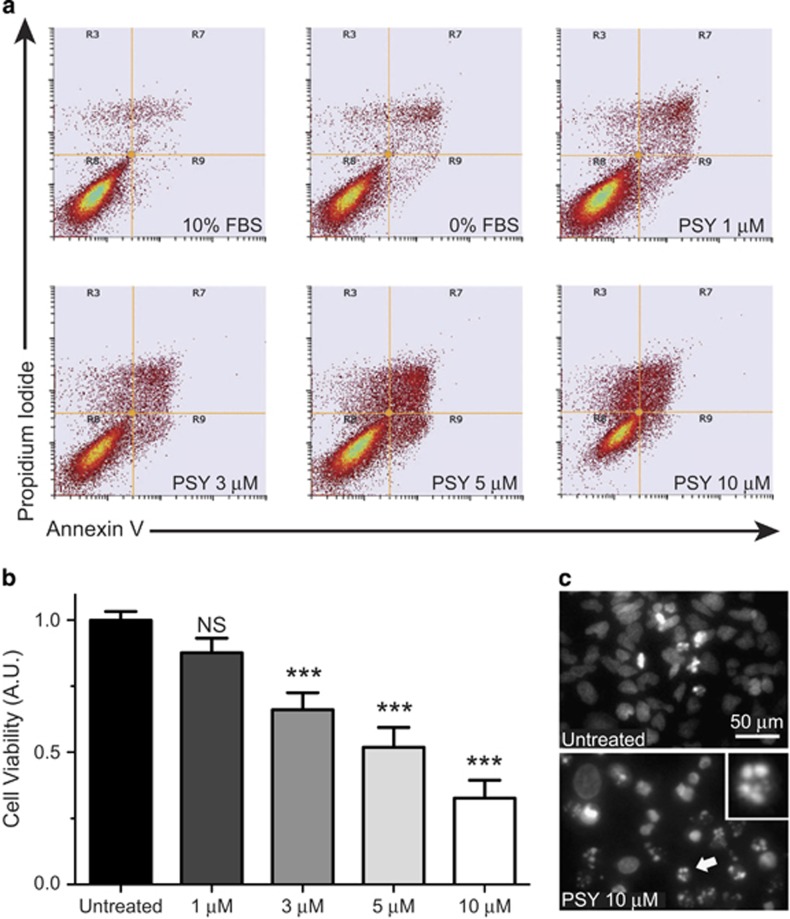
PSY induces dose-dependent cell death in MO3.13 cell line. (**a**) Representative dot-plots of MO3.13 cells stained with Annexin V/propidium iodide and analyzed by flow cytometry: R8, R9, R7 and R3 quadrants define the healthy, apoptotic, secondary necrotic and necrotic populations, respectively. (**b**) PSY dose–response quantification of cell viability (*n*=7, one-way ANOVA (Dunnett's *post-hoc* test ) PSY 1–10 *μ*M *versus* Untreated). (**c**) Representative nuclear morphologies (imaged by Hoechst staining and fluorescence microscopy) for Untreated (top panel) and PSY-10-*μ*M-treated (bottom panel) cells. The bottom panel inset shows a zoomed image of the nucleus indicated by the white arrow. The presence of condensed/fragmented nuclei is a typical feature of apoptosis induction

In order to evaluate PSY effects on cell viability, MO3.13 cells were treated for 24 h with different concentrations of PSY (1–10 *μ*M) in serum-free condition to exclude the sphingolipid content present in serum (as sphingosine 1-phosphate (S1P)),^19^ which could have hindered the PSY effect. After treatment, cells were harvested and stained with Annexin V-FITC conjugate and propidium iodide (PI), and analyzed by flow cytometry (Figure 1a).

Although only slightly reducing cell viability (Supplementary Figure S1a), serum starvation led to MO3.13 cell differentiation toward a more mature oligodendrocyte phenotype, as revealed by the marked change in cell morphology and the enhanced expression of myelin basic protein (Supplementary Figure S1b). As expected, PSY administration induced a dose-dependent cell death that started to be statistically significant from the concentration of 3 *μ*M. Specifically, cell viability was reduced by 34±6% (*P*<0.001), 48±7% (*P*<0.001) and 67±7% (*P*<0.001) for 3 *μ*M, 5 *μ*M and 10 *μ*M PSY concentration, respectively (Figure 1b). Cell death occurred essentially by apoptosis, as demonstrated by the high percentage of Annexin V-positive cells and by the significant presence of condensed and fragmented nuclear morphologies (Figure 1c). The presence of a minor population of cells positive only for PI (Figure 1a, R3 quadrants) indicates that PSY also induced necrotic cell death.

### PSY causes cytoplasmic and mitochondrial Ca^2+^ increases

Because Ca^2+^ is well recognized to be an important messenger during apoptosis, we measured its dynamics upon PSY administration by fluorescence confocal microscopy in living MO3.13 cells. The cells were co-stained with the calcium dye Fluo-3 AM and with the mitochondrial potential dye tetra-metyl rhodamine (TMRM) to also monitor mitochondrial potential loss during the last phases of apoptosis.^20^ The stained cells were treated for 6–8 h with PSY 10 *μ*M and imaged by time-lapse confocal microscopy to follow the cytoplasmic Ca^2+^ kinetics from PSY administration (*t*=0) until cell death occurred.

**Figure 2:**
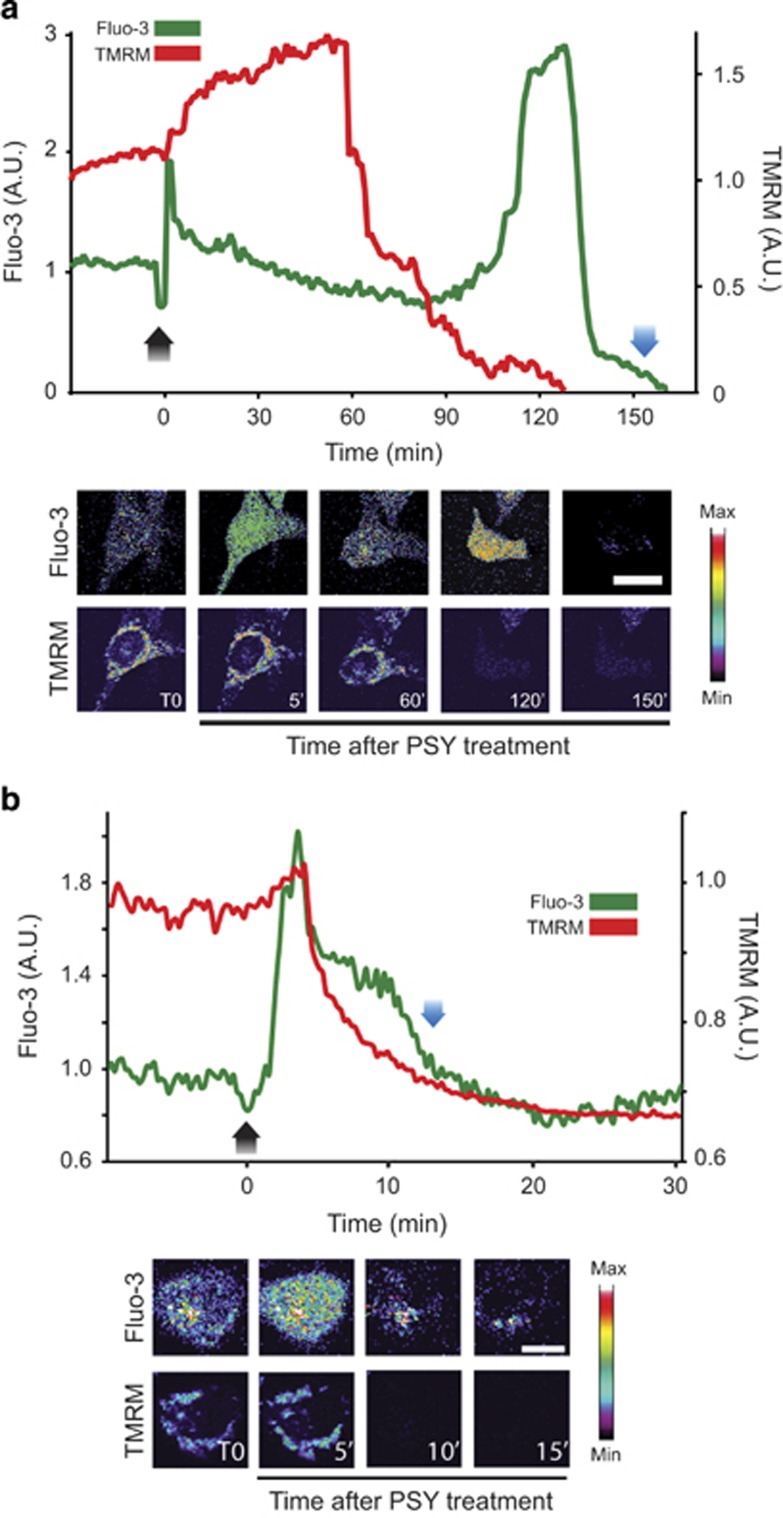
PSY induces cytoplasmic Ca^2+^ increases and mitochondrial depolarization. Representative traces of cytoplasmic Ca^2+^ (green lines) and mitochondrial potential (red lines) kinetics upon PSY 10 *μ*M administration (*t*=0; black arrows) (*n*=3, >25 analyzed cells). Fluo-3 and time-lapse fluorescence imaging of representative cells are reported below the traces. (**a**) Apoptotic Ca^2+^ dysregulation: PSY induces a first Ca^2+^ peak (*t*=5–8 min), and a second, more protracted, Ca^2+^ elevation before cell lysis (blue arrow). The mitochondrial potential shows a first hyper-polarization followed by progressive leak until cell lysis. (**b**) Necrotic Ca^2+^ dysregulation: only the first Ca^2+^ peak is present, coupled with fast mitochondrial depolarization until cell lysis that, in this case, occurred at *t*=13 min

**Figure 3:**
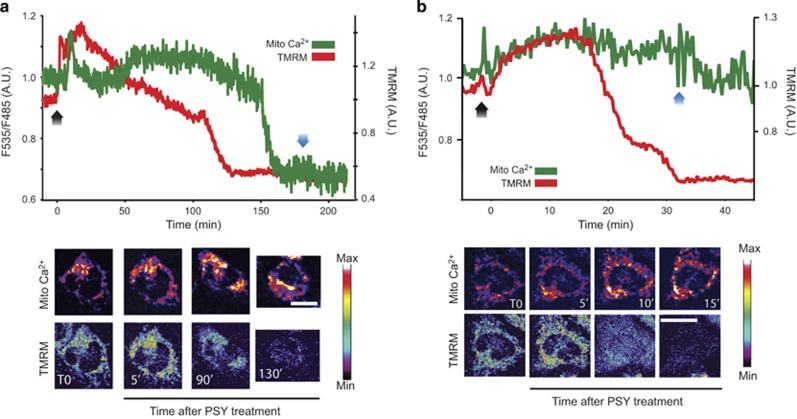
PSY induces mitochondrial Ca^2+^ elevations. Representative traces of Mitochondrial Ca^2+^ (green lines) and potential (red lines) kinetics upon PSY 10 *μ*M administration (*t*=0; black arrows) (*n*=3, >15 analyzed cells). Time-lapse images of the MTCD2CPV FRET signal and TMRM fluorescence of representative cells are reported below the traces. (**a**) Apoptotic Ca^2+^ dysregulation: PSY induces a first mitochondrial Ca^2+^ peak (*t*=5–8 min), and a second, more protracted, Ca^2+^ elevation before cell lysis (blue arrow). The mitochondrial potential shows a first hyperpolarization followed by progressive leak until cell lysis. (**b**) Necrotic Ca^2+^ dysregulation: only the first Ca^2+^ peak is detectable, followed by a progressive Ca^2+^ increase coupled with fast mitochondrial depolarization until cell lysis that, in this case, occurred at *t*=30 min

In the majority of the cells, PSY induced a first rapid Ca^2+^ peak after treatment (*t*≈5–8 min), followed by a second, slower Ca^2+^ increase that preceded cell death (Figure 2a). PSY acted also on mitochondrial potential causing a hyperpolarization followed by a progressive potential leak that ran out with TMRM loss of fluorescence (Figure 2a). During this phase, cells showed an evident membrane blebbing, typical hallmark of apoptosis execution. Among the cells that died by apoptosis, the lifetime distribution within the time-lapse interval (*t*≤8 h from PSY administration) showed two peaks (Supplementary Figure S2), suggesting the presence of two cell sub-populations with different tolerance to PSY. In particular, a group of cells had an average survival time of 35±2 min, whereas the other one, more resistant to PSY, died at *t*=165±11 min after treatment. Both these populations were characterized by a protracted phase of calcium dysregulation (Figure 2a). Differently, a minor population of MO3.13 cells reacted to PSY with a very fast Ca^2+^ increase coupled to mitochondrial potential loss, followed by cell lysis without membrane blebbing, indicating necrotic cell death induction (Figure 2b). These cells were extremely sensitive to PSY and died soon after treatment with an immediate calcium dysregulation.^21^ Control experiments were performed by treating cells with the same quantity of solvent (DMSO) and the cells failed to show any significant change in calcium or mitochondrial potential with respect to the baseline, demonstrating the specificity of PSY effects (Supplementary Figure S3a). Moreover, in order to demonstrate that TMRM fluorescence was correctly correlated to mitochondrial potential in our experimental set-up, we performed control experiments where TMRM signal variations were measured upon uncoupler (carbonyl cyanide m-chlorophenyl hydrazine, 25 *μ*M) treatment (Supplementary Figure S8). This measure confirmed fluorescence-relative variations of the same order as those reported in Figures 2 and 3.

In order to evaluate whether mitochondria could be involved in the observed cytoplasmic Ca^2+^ increase following PSY treatment, MO3.13 cells were transfected with the MTCD2CPV mitochondrial Ca^2+^ FRET probe (see section 'Transfections' for details). We first verified the correct probe localization inside mitochondria: to this end, MTCD2CPV-transfected cells were stained with TMRM and analyzed by high-resolution confocal microscopy. High-magnification images were acquired along the *z* axis by sequential acquisitions of the FRET signal and the TMRM fluorescence to avoid cross talking between TMRM and YFP excitation. Experiments confirmed the co-localization of the MTCD2CPV probe with TMRM (Supplementary Figure S4). Next, we performed time-lapse experiments with MTCD2CPV-expressing cells stained with TMRM. TMRM fluorescence loss was used as time reference to compare mitochondrial Ca^2+^ recordings and the experiments performed with Fluo-3, with the aim to understand whether the cytoplasmic Ca^2+^ kinetics was correlated to mitochondrial Ca^2+^ variations. After at least 30 min of baseline recording, we treated cells with PSY 10 *μ*M. PSY caused a mitochondrial Ca^2+^ increase that well correlated with the PSY-induced cytoplasmic Ca^2+^ increase. Indeed, PSY induced a rapid mitochondrial Ca^2+^ peak (*t*≈5–8 min), followed by recovery to baseline levels and by a second more protracted Ca^2+^ elevation. This last event started before the onset of mitochondrial potential decrease, and was maintained until cell death occurred (Figure 3a). Similarly to what observed for cytoplasmic Ca^2+^ kinetics, in the subset of PSY-treated cells undergoing necrosis, we measured a single mitochondrial Ca^2+^ increase and fast mitochondrial potential loss, followed by membranes lysis with no blebbing (Figure 3b). Control experiments were performed by treating cells with the same quantity of solvent (DMSO) and the cells failed to show relevant changes in mitochondrial Ca^2+^ or potential with respect to the baseline, demonstrating the specificity of PSY effects (Supplementary Figure S3b).

### PSY induces intra-mitochondrial ROS production

**Figure 4:**
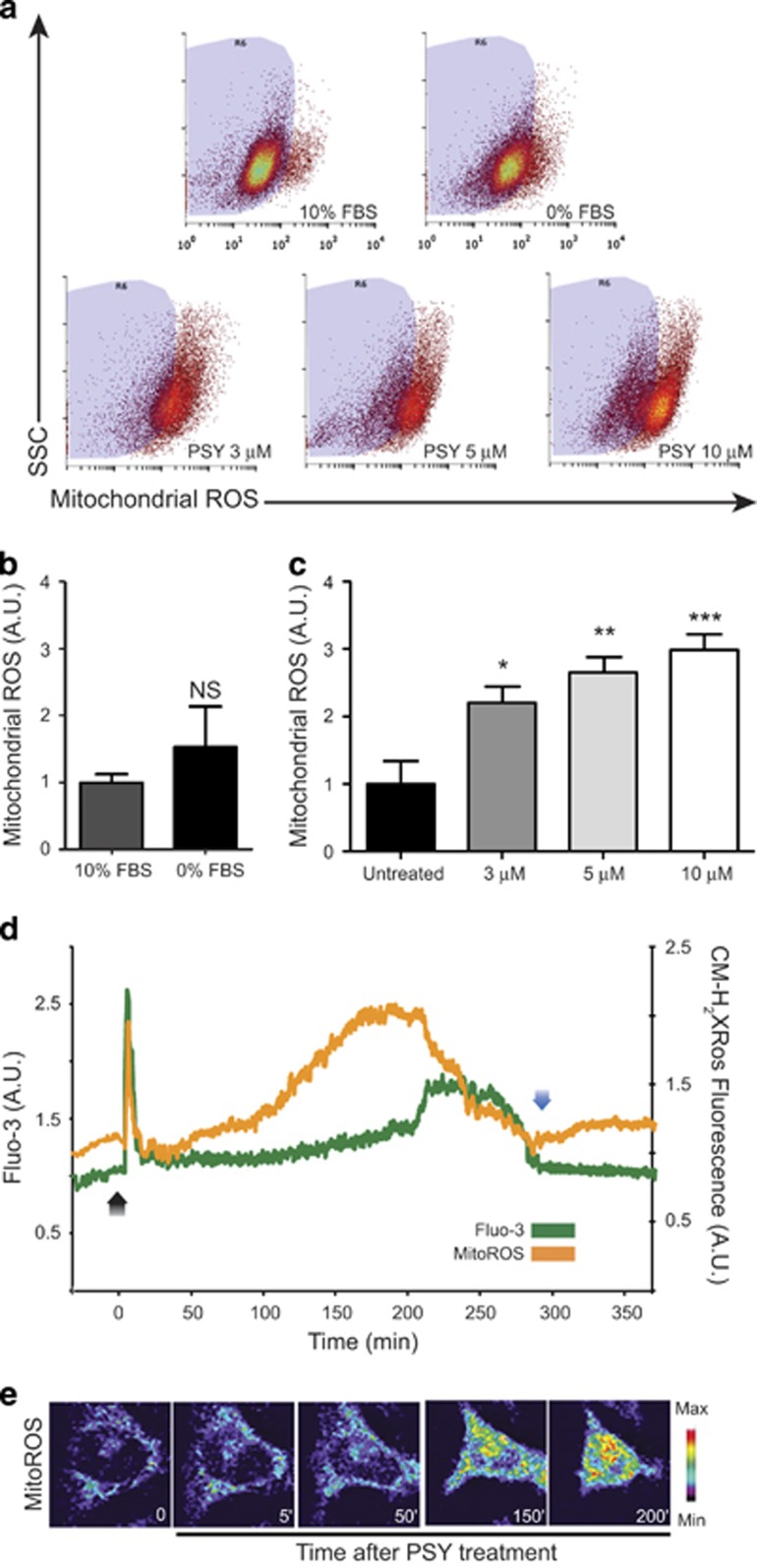
PSY induces mitochondrial ROS production. (**a**) Representative dot-plots of MO3.13 cells stained with Mitotracker Red CM-H_2_XRos and analyzed by flow cytometry. The side-scattered light signal (SSC) is also reported. The R6 region was set by the dot-plot obtained for the control population in (no PSY, 10% FBS). (**b**) Mitochondrial ROS production in case of cell starvation (0% FBS) and culture maintenance condition (10% FBS). (*n*=6, *t*-test). (**c**) PSY dose–response quantification of mitochondrial ROS production (*n*=6, one-way ANOVA (Dunnett's *post-hoc* test ) PSY 3–10 *μ*M *versus* Untreated). (**d**) Representative traces of mitochondrial ROS (orange line) and cytoplasmic Ca^2+^ (green line) kinetics upon PSY 10 *μ*M administration (*t*=0; black arrow) during apoptotic cell death (*n*=3, >15 analyzed cells). PSY induced a first, rapid ROS production peak correlated with the first Ca^2+^ peak; a second, more protracted, mitochondrial ROS elevation was recorded before cell lysis (blue arrow). (**e**) Time-lapse images of the Mitotracker Red CM-H_2_XRos fluorescence of a representative cell is reported below the traces

As our experiments showed a clear PSY effect on mitochondrial Ca^2+^ and because Ca^2+^ increase inside mitochondria can induce ROS production, we evaluated whether these two events were coupled during PSY-induced cell death. To this end, we treated MO3.13 for 24 h with PSY 3–10 *μ*M; after treatments, cells were stained with MitoTracker Red CM-H_2_XRos and measured by flow cytometry (Figure 4a) as described in Materials and Methods.

Figure 4b shows that serum deprivation did not induce a significant increase of mitochondrial ROS; conversely, all PSY concentrations induced relevant mitochondrial ROS production, as we hypothesized (Figure 4c). The maximum oxidative stress was measured for PSY 10 *μ*M, which led to about a three folds of increase with respect to the value of control cells in 0% fetal bovine serum (*P*<0.001).

As flow cytometry gives an averaged picture after 24 h of treatment, time-lapse experiments by confocal microscopy were carried out to characterize the kinetics of this PSY-induced mitochondrial ROS production. MO3.13 cells were stained with Fluo-3 and MitoTracker Red CM-H_2_XRos to monitor both cytoplasmic calcium and ROS production. PSY induced an early peak in CM-H_2_XRos fluorescence soon after administration; this peak corresponded with the early calcium peak detected by Fluo-3 fluorescence. After this peak, the signal gradually increased reaching its maximum before a second cytoplasmic Ca^2+^ increase and indicating robust ROS elevation for 50 min<*t*<200 min (Figure 4d). This result confirms data obtained by flow cytometry. Moreover, we notice that this cytoplasmic Ca^2+^ dynamics was qualitatively the same as that reported in the previous section and that CM-H_2_XRos fluorescence was closely correlated with cytosolic Ca^2+^ elevations. Control experiments were performed by treating cells with the same quantity of solvent (DMSO) and the cells failed to show any important change in CM-H_2_XRos fluorescence or mitochondrial potential with respect to the baseline, demonstrating the specificity of PSY effects (Supplementary Figure S3c).

### Reducing extracellular Ca^2+^ influx extends cell survival and reduces mitochondrial ROS production

Following the observation that PSY treatment triggers calcium elevations, we wondered whether Ca^2+^ influx from the extracellular environment is causally involved in PSY-induced cell death.

**Figure 5:**
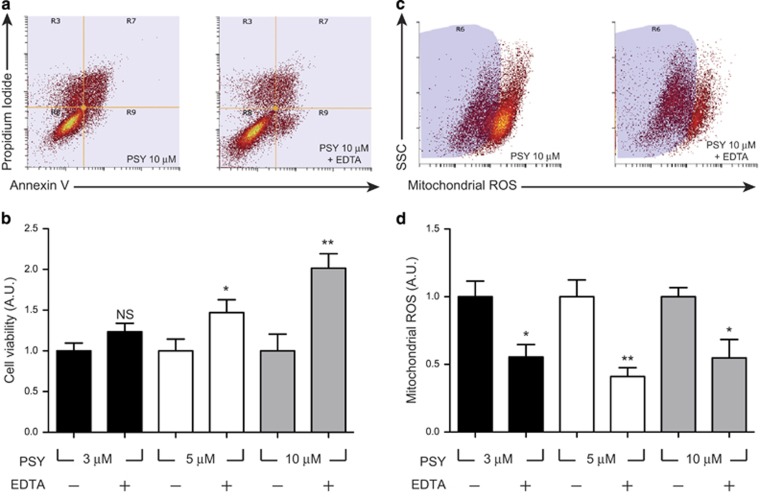
EDTA treatment extends cell survival and reduces mitochondrial ROS production. (**a**) Representative dot-plots of MO3.13 cells treated with PSY 10 *μ*M or with PSY 10 *μ*M in presence of EDTA 1 mM, stained with Annexin V/propidium iodide and analyzed by flow cytometry: R8, R9, R7 and R3 quadrants define the healthy, apoptotic, secondary necrotic and necrotic populations, respectively. (**b**) Cell viability quantification of the flow cytometry dot-plots for cells treated with PSY 3–10 *μ*M, with (+) of without (−) EDTA 1 mM administration (*n*=7, *t*-test EDTA (−) *versus* EDTA (+) for same PSY concentrations). (**c**) Representative dot-plots of MO3.13 cells treated with PSY 10 *μ*M or with PSY 10 *μ*M in presence of EDTA 1 mM, stained with Mitotracker Red CM-H_2_XRos and analyzed by flow cytometry. The side-scattered light signal (SSC) is also reported. The R6 region was set by the dot-plot obtained for the control population in (no PSY, 10% FBS). (**d**) Mitochondrial ROS production quantification of the flow-cytometry dot-plots for cells treated with PSY 3–10 *μ*M, with (+) of without (−) EDTA 1 mM administration (*n*=4, *t*-test EDTA (−) *versus* EDTA (+) for same PSY concentrations)

In order to reduce calcium influx from the medium, extracellular Ca^2+^ content was reduced by using a chelating agent ethylenediaminetetraacetic acid (EDTA). Initially, we performed preliminary dose–response experiments to determine the maximal EDTA concentration not affecting cell viability or ROS production. On the basis of the data reported in Supplementary Figures S5 and S7, we chose 1 mM as the concentration for experiments in the presence of PSY, corresponding to 62% Ca^2+^ reduction in our cell-culture medium (see Materials and Methods for details). MO3.13 cells were treated with PSY 3–10 *μ*M in presence of EDTA for 24 h, then flow cytometry was carried out to assess viability as previously described (see 'PSY induces apoptotic and necrotic cell death' and Materials and Methods). Our data demonstrate that EDTA can protect MO3.13 from PSY-induced cell death. Indeed, EDTA increased cell viability 1.5-fold in case of PSY 5 *μ*M, and twofold in case of PSY 10 *μ*M with respect to cells treated with same concentrations of PSY but without EDTA (Figures 5a and b).

Given this protective effect, we checked whether mitochondrial ROS production was affected by the reduction of calcium influx. Parallel experiments were carried out treating the cells with PSY 3-10 *μ*M in the presence of EDTA for 24 h, and then staining with MitoTracker ROS CM-H_2_XRos for flow cytometry analysis. Experiments showed that EDTA could significantly reduce mitochondrial ROS production for all the tested PSY concentrations, demonstrating that nearly the 50% of mitochondrial ROS production can be prevented by simply reducing extracellular Ca^2+^ concentration (Figures 5c and d).

### Pharmacological reduction of calcium influx and ROS production by EDTA and N-acetyl-cysteine (NAC) had no synergistic effects on cell survival

Finally, as our data showed that EDTA protection led also to the reduction of mitochondrial ROS, we tested whether a treatment reducing both calcium influx and mitochondrial ROS could produce synergistic effects on cell survival after PSY administration.

**Figure 6:**
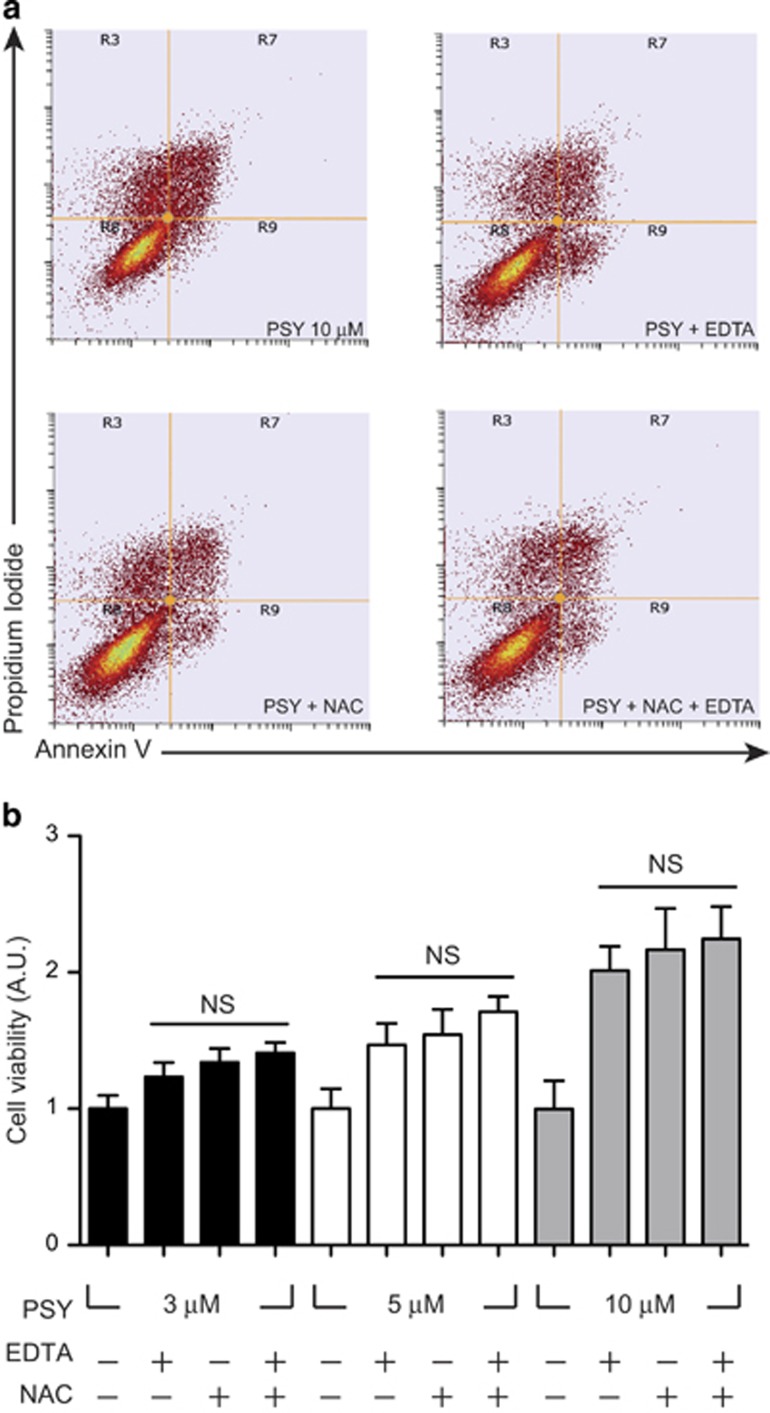
Pharmacological reduction of calcium influx and ROS production by EDTA and N-acetyl-cysteine has no synergistic effect on cell survival. (**a**) Representative dot-plots of MO3.13 cells treated with PSY 10 *μ*M alone, or in combination with EDTA 1 mM, NAC 5 mM and EDTA 1 mM+NAC 5 mM, stained with Annexin V/propidium iodide and analyzed by flow cytometry: R8, R9, R7 and R3 quadrants define the healthy, apoptotic, secondary necrotic and necrotic populations, respectively. (**b**) Cell viability quantification of the flow cytometry dot-plots for cells treated with PSY 3–10 *μ*M, with (+) of without (−) EDTA 1 mM and NAC 5 mM administration. (*n*=5, one-way ANOVA, (Tukey post-hot test): EDTA(+)/NAC(−) *versus* EDTA(−)/NAC(+) *versus* EDTA(+)/NAC(+) for PSY 3 *μ*M and PSY 10 *μ*M; one-way ANOVA, (Dunns *post-hoc* test) EDTA(+)/NAC(−) *versus* EDTA(−)/NAC(+) *versus* EDTA(+)/NAC(+) for PSY 5 *μ*M)

In order to test this hypothesis, MO3.13 cells were treated with PSY 10 *μ*M for 24 h in the presence of the antioxidant NAC 5 mM. This concentration was chosen according to previous results present in the literature.^10^ Cells were harvested and stained with Mito tracker ROS CM-H2XRos and then analyzed by flow cytometry (Figures 6a and S7). As expected, NAC reduced mitochondrial ROS production; the Mito tracker ROS fluorescence was almost halved with respect to all the samples with same concentrations of PSY but without NAC (Supplementary Figure S6a,b). NAC treatment also significantly inhibited PSY-induced MO3.13 cell death as revealed by the Annexin V/PI experiments; differences were statistically significant for PSY 10 *μ*M (Supplementary Figure S6c,d). However, when we measured the viability of MO3.13 cells treated with PSY for 24 h in the presence of both EDTA (1 mM) and NAC (5 mM), we did not observe any significant difference with respect to cells treated with EDTA or NAC alone, indicating that these drugs had no synergistic effect on cell survival after PSY treatment (Figures 6b and S7).

## Discussion

GLD is a neurodegenerative disease characterized by the intracellular accumulation of PSY, a cytotoxic sphingolipid. It has been hypothesized that PSY has a crucial role in dysfunction of myelinating cells and white matter loss, but little is known about its specific intracellular effects. The aim of this work is to shed light on specific PSY-induced intracellular pathways that are currently incompletely understood, and that could be impacted by pharmacological treatments with the aim to possibly delay GLD onset and/or slow down its progression.

It is well known that several sphingolipid messengers such as ceramide, glucosyl-ceramide, ceramide-1-P, sphingosine and sphingosine-1-phosphate can regulate intracellular Ca^2+^ homeostasis.^22, 23, 24, 25, 26^ Likewise, few studies in the 90s showed that PSY can mobilize nuclear and cytoplasmic Ca^2+^ in isolated rat liver nuclei ^27^ and in several cultured cell lines (e.g., MC3T3-E1 pre-osteoblasts,^28^ Jurkat T-cells, RINm5F Insulinoma cells^29^), respectively. More recently, two papers by Lloyd-Evans *et al.*^30, 31^ reported Ca^2+^ release in rat brain microsomes upon PSY treatment, with PSY acting as an agonist of ryanodine receptors. Interestingly, these references documented relevant Ca^2+^ alterations (as increase in cell cytoplasm and insolated nuclei, and release form microsomes) within ≈10 min from PSY (up to 30 *μ*M) administration, but Ca^2+^ kinetics on longer time-scales was not investigated. Moreover, in contrast with other lysosomal storage disorders, no clear evidence has been still reported that Ca^2+^ dysregulation is a pathogenic factor in GLD.^32^ Our data are full in agreement with the above-presented literature, extending the characterization of Ca^2+^ dynamics until PSY-induced cell death occurs; PSY accumulation is indeed believed to be the main cause of oligodendrocyte loss in GLD. Moreover, in order to make our analysis more relevant for shedding light on GLD pathogenesis, we used a differentiated human oligodendrocyte cell line (starved MO3.13 cells) for all the experiments. MO3.13 cells are indeed widely used for GLD research (see, e.g., Won *et al.*^33^ and Giri *et al.*^8^) because of their very active sphingolipid metabolism similar to that of primary oligodendrocytes.^34^ Our experiments of Ca^2+^ chelation directly support the hypothesis that Ca^2+^ dysregulation by PSY can trigger oligodendrocyte loss.

By treating MO3.13 cells with exogenous PSY, we observed necrotic and apoptotic cell death, as expected. The necrotic population died by immediate calcium dysregulation (Figure 2b), whereas the apoptotic population died by delayed calcium dysregulation (Figure 2a).^21^ In the apoptotic population, after the mentioned early Ca^2+^ peak occurring few minutes after treatment, the cytoplasmic Ca^2+^ presented a second robust elevation during the last phases of apoptosis execution (Figure 2a). These sustained unbalances in intracellular Ca^2+^ might originate from the activation of specific membrane channels ^35, 36, 37^ or from a direct permeabilization of the plasma membrane owing to the change in its lipid composition. Structural sphingolipids such as galactosil-ceramide, one of the substrate of the GALC enzyme, are localized in membrane microdomains called rafts. Rafts are membrane regions enriched in sphingolipids and cholesterol: this composition makes raft structures less fluid than the surrounding phospholipids bilayer, and for this reason, they are involved in cell shaping, cytoskeleton anchoring to the plasma membrane, trafficking of proteins and receptors clustering or regulation.^38, 39, 40^ It has been recently demonstrated that PSY specifically accumulates in membrane microdomains in the brain of Twitcher mice and GLD patients, disrupting the raft architecture. Sub-cellular raft fractions resulted enriched in cholesterol as well, indicating a change in the overall raft composition during GLD progression.^4^ Rafts are tightly linked to Ca^2+^ regulation: the activation of store-operated Ca^2+^ influx is modulated by lipid rafts, and the transient receptor channels TRPC1 and TRPC6 are involved in raft-mediated Ca^2+^ influx.^41^ A possible scenario is that the PSY-induced raft disruption can also change the overall membrane Ca^2+^ permeability and/or determine an active Ca^2+^ influx mediated by channels anchored to lipid rafts.

Our data demonstrate that the cytoplasmic Ca^2+^ increase induced by PSY is accompanied by an increase of mitochondrial Ca^2+^. Mitochondria are organelles that can locally sense the cytoplasmic Ca^2+^ and are localized in specific subcellular zones where their activity is required. In basal conditions, mitochondrial Ca^2+^ is maintained at low concentrations (≈100 nM), but mitochondria can sense cytoplasmic Ca^2+^ variations and transiently sustain high Ca^2+^ concentrations (hundreds of *μ*M),^42^ thus contributing to Ca^2+^ homeostasis in the cell. Slight mitochondrial Ca^2+^ increases can regulate the mitochondrial activity by activating the Krebs cycle dehydrogenases, by promoting the supply of oxidable substrates and by regulating the activity of ATP synthase.^16^ This is one of the mechanisms used by cells to reduce the cytoplasmic Ca^2+^, because ATP production can stimulate the sarco endoplasmic calcium ATPase that transfers Ca^2+^ from the cytoplasm to the endoplasmic reticulum.^43^ We reported a progressive increase of the mitochondrial potential during the first phases following PSY administration, indicating stimulation of mitochondrial activity and suggesting the activation of the endoplasmic reticulum for storing the Ca^2+^ excess. Conversely, robust mitochondrial Ca+ elevations lead to ROS production that runs out with mitochondrial membranes destruction, release of cytochrome C and apoptosis induction.^16^ Following the first Ca^2+^ peak, we indeed measured a second, sustained Ca^2+^ increase, and enhancement of mitochondrial ROSs (Figure 4d, 50 min<*t*<200 min) before cell death. These data fit with the paradigm of cell death mediated by mitochondrial Ca^2+^ increase and ROS production, as previously hypothesized by Formichi and co-workers.^7^ Interestingly, mitochondrial abnormalities, such as loss of mitochondrial Ca^2+^ buffering and malfunctioning in the lysosomal-mitochondrial axis, have been also reported in other lysosomal storage disorders.^44^

Therefore, given this evidence that PSY can induce a protracted Ca^2+^ elevation and mitochondria-mediated cell death, we hypothesized that protection from PSY cytotoxicity could result from reducing the extracellular calcium content and mitochondrial oxidative stress, possibly in a synergic way. We found that Ca^2+^ chelation from the extracellular medium by EDTA (1 mM) improves cell survival following PSY administration (Figure 5b) and reduces mitochondrial ROS production by 50% (Figure 5d). Similarly, halving ROS production by NAC also results in enhancements of cell viability (Supplementary Figure S6). We finally treated cells with both EDTA and NAC, and we obtained no synergic effect on cell viability (Figure 6).

This finding suggests that Ca^2+^ influx might be the main cause of mitochondrial ROS production, acting upstream of the mitochondrial oxidative stress induction. Yet, we cannot exclude that the concentrations used for EDTA and NAC were high enough to independently saturate cell protection mechanisms, thus hindering the observation of a possible cumulative effect.

The demonstration of the role of calcium in PSY-induced cell death gives some indications on possible therapeutic targets to be tested in GLD models, such as the raft-associated calcium channels (e.g., the TRPC). Moreover, given the important role of mitochondrial ROS production, antioxidants that specifically target mitochondria might be tested. The MitoQ, for example, is a mitochondria-targeted antioxidant designed to accumulate within mitochondria *in vivo* in order to protect against oxidative damage. This molecule has already undergone clinical trials in humans, showing optimal results in terms of safety for up to 1 year of treatment.^45^

## Conclusions

In this study, we have followed longitudinally calcium dynamics and mitochondrial ROS during PSY-induced oligodendrocyte cell death. We report that PSY treatment causes elevations of cytosolic and mitochondrial Ca^2+^ that are coupled to mitochondrial ROS production. Chelation of extracellular calcium decreases intra-mitochondrial ROS production and enhances cell viability. Antioxidant treatment also reduces mitochondrial ROS production and cell loss, but this therapy does not synergize with Ca^2+^ chelation. Altogether, these data provide novel information on the intracellular pathways activated during PSY-induced toxicity.

## Materials and Methods

### Cell culture and treatments

Human oligodendrocyte MO3.13 cells (Tebu Bio, Le-Perray-en-Yvelines, France, Cat. No. CLU301-P) were maintained in DMEM medium supplemented with 2 mM L-glutamine, 1% penicillin/streptomicyn and 10% heat-inactivated fetal bovine serum (GIBCO-Life Technologies, Carlsbad, CA, USA), at 37 °C in humidified atmosphere containing 5% CO2.

MO3.13 cells were seeded at 25000 cells/cm^2^; 24 h after plating, the medium was removed and cells were washed two times with phosphate-buffered saline (PBS) 1 × . Then, cells were cultured in serum-free medium (DMEM, supplemented with 2 mM L-glutamine, 1% penicillin/streptomicyn) or serum-free medium+PSY 1–10 *μ*M. For EDTA and NAC experiments, cells were pre-treated for 30 min with EDTA 1 mM or/and NAC 5 mM, then treated with PSY 3–10 *μ*M for 24 h. PSY was diluted in DMSO and prepared as a 10 mM stock solution, EDTA was diluted in water and prepared as 1 M stock solution and NAC was diluted in water as 1 M stock solution; control cultures received the same quantity of vehicle (DMSO or/and water). Given the concentrations of Ca^2+^ and Mg^2+^ in the culture medium (1.8 mM and 0.8 mM, respectively, for our DMEM), the binding constants for EDTA with Ca^2+^ and Mg^2+^ (log Ka=10.65 for Ca2+ and 8.79 for Mg^2+^) and the final concentration od EDTA (1 mM), the theoretical amount of free Ca^2+^ and free Mg^2+^ in the medium supplemented with EDTA is calculated to be approximately 0.8 mM for both ions (i.e., in these conditions, essentially no Mg^2+^ is complexed), that is, ≈60% reduction in Ca^2+^ content. We also measured the pH in DMEM and DMEM+EDTA (1 mM) and we did not find important medium acidification (pH from 7.4 to 7.25).

### Annexin V/PI cell death assay

After treatments, MO3.13 cells were harvested and spun for 5 min at 1400 rpm; the obtained pellets were washed with PBS 1 × and spun again for 5 min at 1400 rpm. Cells were re-suspended in binding buffer (10 mmol/l HEPES, 135 mmol/l NaCl, 5 mmol/l CaCl_2_) containing Annexin V-FITC conjugate 1 *μ*M (Invitrogen-Life Technologies, Carlsbad, CA, USA) and PI 100 nM (Sigma-Aldrich, St. Louis, MO, USA) and incubated in ice for 30 min. Flow cytometry was performed by using a S3 flow cytometer (Bio-Rad, Hercules, CA, USA) equipped with 488 and 561 nm diode-pumped solid-state lasers. Annexin V-FITC was excited using the 488 nm laser, and fluorescence was collected through a 552/50 nm band-pass filter and 505 nm long-pass filter; PI was excited with the 561 nm laser and the fluorescence was collected through a 605/40 nm band-pass filter and a 570 nm long-pass fiter. At least 20 000 gated events were acquired for each sample. Data were analysed using the Bio-Rad ProSort software.

### Hoechst staining for condensed nuclei

Cells were fixed with paraformaldeyde 2% for 30 min at room temperature; after fixation, cells were washed once with PBS and stained with Hoechst 33342 (1 *μ*g/ml in PBS, Invitrogen-Life Technologies) for 30 min. Fluorescence and phase contrast images were acquired by using an Eclipse Ti inverted microscope (Nikon, Tokyo, Japan) equipped with a × 20 air Nikon objective, N.A. 0.75, ApoFluor and a CCD ORCA R2 (Hamamatsu, Shizuoka, Japan). Hoechst 33342 was excited by a mercury arc lamp using a 450/50 nm band-pass filter, and emission was collected using an UV-2E/C filter (Nikon). Cell death was qualitatively assessed on the basis of cellular and nuclear morphology evaluating the presence of condensed or fragmented nuclei.

### SDS-PAGE and western blotting

A total of 1 × 10^6^ MO3.13 cells were seeded in 10 cm diameter dishes; 24 h after plating, the medium was removed, cells were washed two times with PBS and incubated for 24 h in serum-free medium, or in medium with 10% fetal bovine serum (control condition for this experiment). Preparation of cell lysates and western blotting were carried out as described in Reimertz *et al.*^46^ The resulting blots were probed with: (i) a rabbit polyclonal anti-myelin based protein antibody (1 : 500, AbCam, Cambridge, UK, ab62631); (ii) a mouse monoclonal anti-tubulin antibody (1 : 50000, Sigma-Aldrich). Horseradish peroxidase-conjugated secondary antibodies (1 : 10000, Pierce, Northumberland, UK) were detected using SuperSignal West Pico Chemiluminescent Substrate (Pierce) and imaged using a ImageQuant LAS-4000 imaging system (GE Healthcare, Little Chalfont, UK).

### Immunofluorescence and confocal analysis

MO3.13 cells were plated on glass cover slips (25000 cells/cm^2^) into 24-well plates for 2 days and then treated with serum-free medium. After 24 h of treatment, cells were fixed for 30 min in 2% paraformaldehyde, permeabilised with 0.1% Tween-20 and blocked with 5% horse serum for 15 min. Cells were then incubated overnight with anti-myelin basic protein antibody (1 : 50, AbCam ab62631). Cover slips were washed with PBS (10 min for three times), incubated with anti-rabbit biotinylated secondary antibody (Jackson Immuno Research Europe, Suffolk, UK, 1 : 500) for 90 min, washed with PBS and then stained with Alexa 488 Streptavidin (Molecular Probes, Netherlands, 1 : 1000) for 30 min. Finally, the cover slips were mounted onto glass microscope slides in presence of Vectashield mounting medium with DAPI (Vector Laboratories inc. Burlingame, CA, USA). Confocal images were acquired as z-stacks by using a scanned on *z* axis with a SP2 TCS-NT Leica (Nussloch, Germany) laser scanning confocal microscope, equipped with 40 × 1.2 NA Plan Apo oil objective. The optical slice was set to 1 *μ*M (FWHM).

### Mitochondrial ROS detection by flow cytometry

MO3.13 cells were seeded in 24 well plates (25000 cells/cm^2^) and treated as described before; after treatments, the cells were stained with MitoTracker Red CM-H_2_XRos (Molecular Probes-Life Technologies, Carlsbad, CA, USA) for 30 min at 37 °C in humidified atmosphere containing 5% CO2. MitoTracker Red CM-H_2_XRos was diluted with anhydrous DMSO to obtain 1 mM of stock solution and used 1 *μ*M as final concentration. After staining, cells were harvested and spun for 5 min at 1400 rpm; the obtained pellets were washed with PBS and spun again for 5 min at 1400 rpm. Flow cytometry was performed by using a S3 flow cytometer (Bio-Rad) equipped with a 488 nm and 561 diode-pumped solid-state lasers. MitoTracker Red CM-H_2_XRos was excited with a 561 nm laser and fluorescence emission was collected through a 605/40 nm band-pass filter and 570 nm long-pass fiter. At least 20 000 gated events were acquired for each sample. Data were analyzed using the Bio-Rad ProSort Software.

### Cytoplasmic Ca^2+^ imaging

MO3.13 cells (50000 cells/cm^2^) were plated in WillCo (Amsterdam, The Netherlands) dishes 24 h before experiments to reach 50–60% of confluence and to obtain a stable substrate adhesion. One hour before the experiment, MO3.13 cells were deprived by serum, stained with Fluo-3 AM 1 *μ*M for 30 min, and then incubated in serum-free medium with TMRM (50 nM). TMRM concentration was chosen to avoid quenching of the dye. One hour later, the WillCo dish was mounted into SP2 TCS-NT Leica laser scanning confocal microscope, equipped with 40 × 1.2 NA Plan Apo oil objective and with an incubating chamber (T=37 °C and 5% CO2). Fluo-3 was excited using the 488-nm laser line, and the emitted fluorescence was collected through a 530/30-nm band-pass filter; TMRM was excited using the 568-nm laser line, and the emitted fluorescence was collected through a 590-nm long-pass filter. Excitatory lights were kept to the minimum power to minimize photodamage and photobleaching. The pinhole of the microscope was set to obtain an optical slice of 0.5 *μ*m and samples were scanned two times at 512 × 512 pixel resolution with a time interval of 15 s. After at least 30 min of baseline recording, PSY 10 *μ*M was added to the medium and cellular response was recorded for 6–8 h.

### Transfections

For mitochondrial calcium measurements, 1 × 10^5^ MO3.13 cells were plated into 1 cm diameter WillCo dishes and, 24 h later, transfected with cDNA encoding for the mitochondrial cameleon FRET probe MTCD2CPV (kindly supplied from Prof. Roger Tsien).^47^ In this construct, a Ca^2+^-responsive element (calmodulin, CaM) is present that, case of Ca^2+^ variations, alters the efficiency of FRET between two fluorescent proteins. The transfection solution was prepared diluting 1 *μ*g of plasmid and 1 *μ*l of Lipofectamine 2000 (Life Technologies, Carlsbad, CA, USA) reagent in OPTIMEM medium (Life Technologies) following the manufacturer's instructions, and incubated at room temperature for 30 min. After liposome reaction, solutions were used to transfect the cells. After 90 min incubation, the transfection medium was removed and replaced with fresh medium. Experiments were performed 72 h after the transfection to have ≈80% of the cells with the correct MTCD2CPV localization into the mitochondria. Ninety minutes before experiments, MO3.13 cells were deprived of serum and after 1 h stained for 1 h with TMRM 50 nM and mounted into the incubator chamber for the acquisition in time-lapse at 37 °C in a humidified atmosphere containing 5% CO2. Images were collected with a time interval of 15 s, with a resolution of 512 × 512 pixels. The MTCD2CPV calcium probe was excited with the 458 laser line of a SP2 TCS-NT Leica confocal microscope and the emissions of CFP and YFP were recorded at 485 and 535 nm, respectively. TMRM was excited with 568-nm laser line in sequential acquisition after cameleon recording to exclude YFP excitation and cross-talking. For each time point, both fluorescent and transmitted light images were acquired. The quantification was performed by using the MetaMorph 5.0 software (Universal Imaging, West Chester, PA, USA) expressing the calcium concentration as ratio of emissions FRET/CFP (535/485). The images were elaborated with the plug-in ‘FRET analyzer' of the Image J free software (http://imagej.nih.gov/ij/).

### Ca^2+^ and mitochondrial ROS evaluation

MO3.13 cells (50000 cells/cm^2^) were plated into WillCo dishes 24 h before experiments to reach 50–60% of confluence and to obtain a stable substrate adhesion. After Fluo-3 AM loading (as described in 'Cytoplasmic Ca^2+^ imaging'), medium was removed and replaced with serum-free medium with MitoTracker Red CM-H_2_XRos 1 *μ*M. Cells were stained for 30 min at 37 °C in a humidified atmosphere containing 5% CO2 and then imaged by using a SP2 TCS-NT Leica confocal microscope, equipped with 40 × 1.2 NA Plan Apo oil objective and with an incubating chamber (T=37 °C, 5% CO2). Fluo-3 signal was recorded as described in 'Cytoplasmic Ca^2+^ imaging'. MitoTracker Red CMH_2_XRos was excited using the 568-nm laser line, and the emitted fluorescence was collected through a 590-nm long-pass filter. Excitatory lights were kept to the minimum power to minimize photodamage and photobleaching. The pinhole of the microscope was set to obtain an optical slice of 0.5 *μ*m and samples were scanned two times at 512 × 512 pixel resolution with a time interval of 15 s. After at least of 30 min of baseline recording, PSY was added to the medium and cells responses was recorded for 6–8 h.

### Statistical analysis

Inferential statistics was used to compare datasets from different experimental groups. All the experiments were repeated at least three times independently (in figure legends, ‘*n*' indicates the number of the performed experiments). Data are reported as the averaged value of the means of single experiments +/− the standard error of the mean (mean +/− S.E.M.). Each distribution of the means or, when required, of full datasets has been tested for normality (e.g., by the Shapiro-Wilk normality test). For parametric data, Student's *t*-test (unpaired, two-tailed) or one-way ANOVA (Tukey's or Dunnett's post tests) were used; for non-parametric data, Mann-Whitney test (two-tailed) or Kruskal-Wallis test was used. Minimum statistical significance will be set for *P* values<0.05 (*P<0.05; **P<0.01; ***P<0.005). NS stands for ‘not significant'.

## Abbreviations

EDTA: ethylenediaminetetraacetic acid
Ca^2+^: calcium
DMSO: dimethyl sulfoxide
DMEM: Dulbecco's Modified Eagle Medium
GALC: galactocerebrosidase
GLD: Globoid cell leukodystrophy
NAC: N-acetyl-cysteine
PI: propidium iodide
PSY: psychosine
ROS: reactive oxygen species
PLA2: secretory phospholipase A2
S1P: sphingosine 1-phosphate
TMRM: tetra-metyl-rhodamine
