# Supplementary material for: Role of extracellular calcium and mitochondrial oxygen species in psychosine-induced oligodendrocyte cell death
Source: Cell Death Dis. 2014 Nov 20;5(11):e1529–. doi: 10.1038/cddis.2014.483 (PMC4260741; doi:10.1038/cddis.2014.483)
Supplement: Supplementary Figures [file cddis2014483x1.doc]

# Supplementary Figures – Voccoli *et al., 2014*

|  |
| --- |
| **Figure S1. MO3.13 cell line characterization.** (a) Cell viability quantification in case of cell starvation (0% FBS) and culture maintenance condition (10% FBS), at t = 24 h. (n=9, Mann-Whitney test). (b) MBP expression increase upon starvation: (top) representative confocal fluorescence images of MO3.13 cells immunostained for MBP (green) and stained with Hoechst 33342 (blue); (bottom) Western Blot quantification of MBP expression (see Materials and Methods for details, Sec. 5.4). Tubulin was used as loading control. Scale Bar = 100 μm. |

|  |
| --- |
| **Figure S2:** **MO3.13 Lifetime distribution** measured during time-lapse experiments upon PSY 10 μm administration (t=0): histogram of cell-death events as a function of time (10-min binning). The histogram has been fitted with a double Gaussian curve (black line); the best fitting parameters are reported in the table. |

|  |
| --- |
| **Figure S3. Representative kinetics control experiments.** Representative traces of (a) cytoplasmic Ca^2+^ (green line) and mitochondrial potential (red line), (b) mitochondrial Ca^2+^ (green line) and mitochondrial potential (red line), and (c) cytoplasmic Ca^2+^ (green line) and mitochondrial ROS (yellow line) upon vehicle (DMSO) administration (blue arrow). |

| 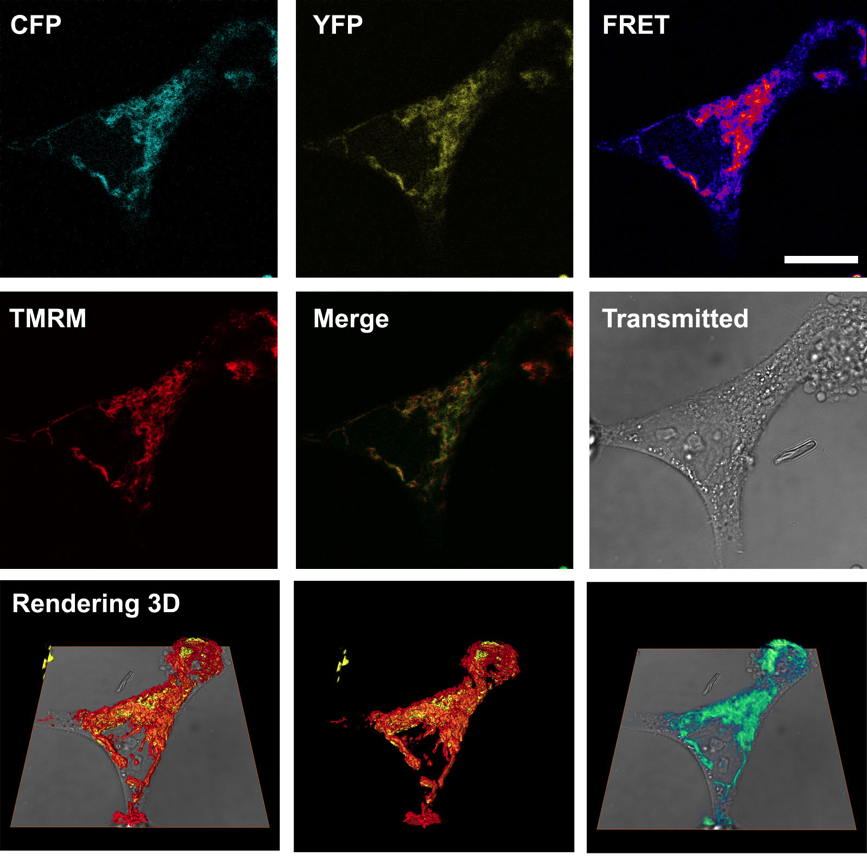 |
| --- |
| **Figure S4. MTCD2CPV localization.** MO3.13 cells transfected with the mitochondrial Ca^2+^ probe MTCD2CPV and stained with TMRM were imaged along the Z-axis every 0.2 μm with a resolution of 1024x1024 pixels. The panel “ merge” shows the good colocalization (yellow) of the MTCD2CPV with mitochondria (TMRM signal). Cells were rebuilt with MetaMorph 5.0 software (Universal Imaging, West Chester, PA) in Z-stacks. After background subtraction a 2 pixel size median filter was applied to the stacks. The 3D rendering of the colocalization map superimposed to the cell bright field image was performed by using Amira software (bottom row). Scale Bar = 20 μm. |

|  |
| --- |
| **Figure S5. EDTA dose response quantification of cell viability.** MO3.13 cells were treated in 0% FBS with EDTA 1mM, 3mM and 5mM for 24 hours and analyzed by AV/PI flow cytometry [n = 3, One-Way ANOVA (Dunnett’s post-hoc test) EDTA 1-5 mM vs Untreated]. |

|  |
| --- |
| **Figure S6. Effect of NAC on Cell Viability and mitochondrial ROS production.** (a) Representative dot-plots of MO3.13 cells treated with PSY 10 μM or with PSY 10 μM in presence of NAC 5mM, stained with Mitotracker Red CMXRos and analysed by flow cytometry. The side-scattered light signal (SSC) is also reported. The R6 region was set by the dot-plot obtained for the control population in (No PSY, 10% FBS). (b) Mitochondrial ROS production quantification of the flow-cytometry dot-plots for cells treated with PSY 3–10 μM, with (+) of without (-) NAC 5 mM administration. [n>3, t-test NAC (-) vs NAC (+) for same PSY concentrations]. (c) Representative dot-plots of MO3.13 cells treated with PSY 10 μM or with PSY 10 μM in presence of NAC 5mM, stained with Annexin V/Propidium Iodide and analysed by flow cytometry: R8, R9, R7 and R3 quadrants define the healthy, apoptotic, secondary necrotic and necrotic populations, respectively. (d) Cell viability quantification of the flow-cytometry dot-plots for cells treated with PSY 3–10 μM, with (+) of without (-) NAC 5 mM administration. [n>3, t-test NAC (-) vs NAC (+) for PSY 3 μM and 10 μM; Mann-Whitney test NAC (-) vs NAC (+) for PSY 5 M]. |

| **(a)** | **(b)** |
| --- | --- |
| **Figure S7. EDTA 1mM and NAC 5mM do not affect cell viability and mitochondrial ROS production.** MO3.13 cells treated with EDTA 1 mM, NAC 5 mM and EDTA 1 mM + NAC 5 mM, stained with Mitotracker Red CMXRos or AV/PI, and analyzed by flow cytometry: (a) mitochondrial ROS production quantification [4 < n < 7, One-Way ANOVA (Dunnet’s post-hoc test EDTA, NAC, EDTA+NAC vs Untreated] and (b) cell viability quantification [n = 3, One-Way ANOVA (Dunnet’s post-hoc test EDTA, NAC, EDTA+NAC vs Untreated]. | |

|  |
| --- |
| **Figure S8. TMRM fluorescence upon CCCP administration.** Representative traces of MO3.13 single-cell TMRM fluorescence kinetics upon CCCP (25 μM, black) and vehicle (gray) administration (t = 0; black arrow). Data from 8 (n=3) and 3 (n=1) cells from independent experiments were collected in single datasets and single-cell fluorescence averaged values were reported with standard deviations as error bars. CCCP was dissolved in EtOH (at 25 mM) and added into Willco dishes to obtain a solvent final concentration of 0.1% in the cell medium. |
